# Regiocontroled Pd-catalysed C5-arylation of 3-substituted thiophene derivatives using a bromo-substituent as blocking group

**DOI:** 10.3762/bjoc.12.210

**Published:** 2016-10-17

**Authors:** Mariem Brahim, Hamed Ben Ammar, Jean-François Soulé, Henri Doucet

**Affiliations:** 1 Institut des Sciences Chimiques de Rennes, UMR 6226 CNRS-Université de Rennes "Organométalliques: Matériaux et Catalyse", Campus de Beaulieu, 35042 Rennes, France. Tel.: 00-33-2-23-23-63-84; 2 Laboratoire de Synthèse Organique Asymétrique et Catalyse Homogène, (UR 11ES56) Université de Monastir, Faculté des Sciences de Monastir, avenue de l’environnement, Monastir 5000, Tunisia

**Keywords:** aryl bromides, C–H bond activation, catalysis, direct arylation, palladium, thiophenes

## Abstract

The use of a bromo-substituent as blocking group at the C2-position of 3-substituted thiophenes allows the regioselective introduction of aryl substituents at C5-position via Pd-catalysed direct arylation. With 1 mol % of a phosphine-free Pd catalyst, KOAc as the base and DMA as the solvent and various electron-deficient aryl bromides as aryl sources, C5-(hetero)arylated thiophenes were synthesized in moderate to high yields, without cleavage of the thienyl C–Br bond. Moreover, sequential direct thienyl C5-arylation followed by Pd-catalysed direct arylation or Suzuki coupling at the C2-position allows to prepare 2,5-di(hetero)arylated thiophenes bearing two different (hetero)aryl units in only two steps. This method provides a “green” access to arylated thiophene derivatives as it reduces the number of steps to prepare these compounds and also the formation of wastes.

## Introduction

**Figure 1:**
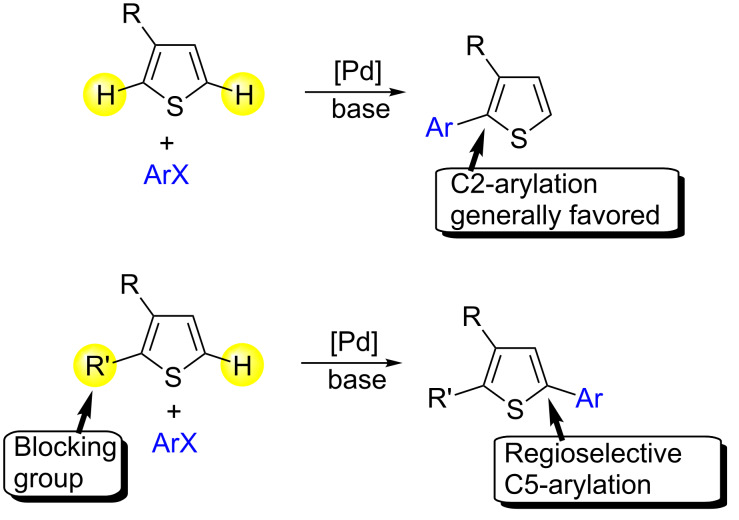
Regioselectivity of the arylation of 3-substituted thiophenes.

Thiophene derivatives bearing aryl substituents are important structures because of their biological and/or physical properties. Among them, 3-substituted 5-arylthiophenes are widely used as building blocks for the synthesis of semi-conductors [1–3]. Therefore, the discovery of more direct and selective procedures for access to 5-arylated 3-substituted thiophene derivatives is an important topic in sustainable chemistry [4]. Stille or Suzuki palladium-catalysed coupling reactions [5–10] are some of the most efficient methods for the preparation of 5-arylated 3-substituted thiophenes [11–14]. However, before these coupling reactions can be performed, an organometallic compound must be synthesized. In 1990, Ohta and co-workers described the Pd-catalysed direct arylation of thiophene derivatives by coupling reaction with aryl halides [15–16]. This is a highly powerful method for a greener access to a very broad range of arylated thiophenes [17–25]. The method is very attractive in terms of green chemistry, because its major by-products are not metal salts but a base associated to HX, and synthesis of an organometallic derivative can be avoided. However, for C3-substituted thiophenes, arylation generally occurred at the C2-position or gave mixtures of C2- and C5-arylated products [26–33]. The introduction of blocking groups at C2-position on thiophene derivatives in order to arylate regiospecifically the C5-positions had been reported (Figure 1).

**Scheme 1:**
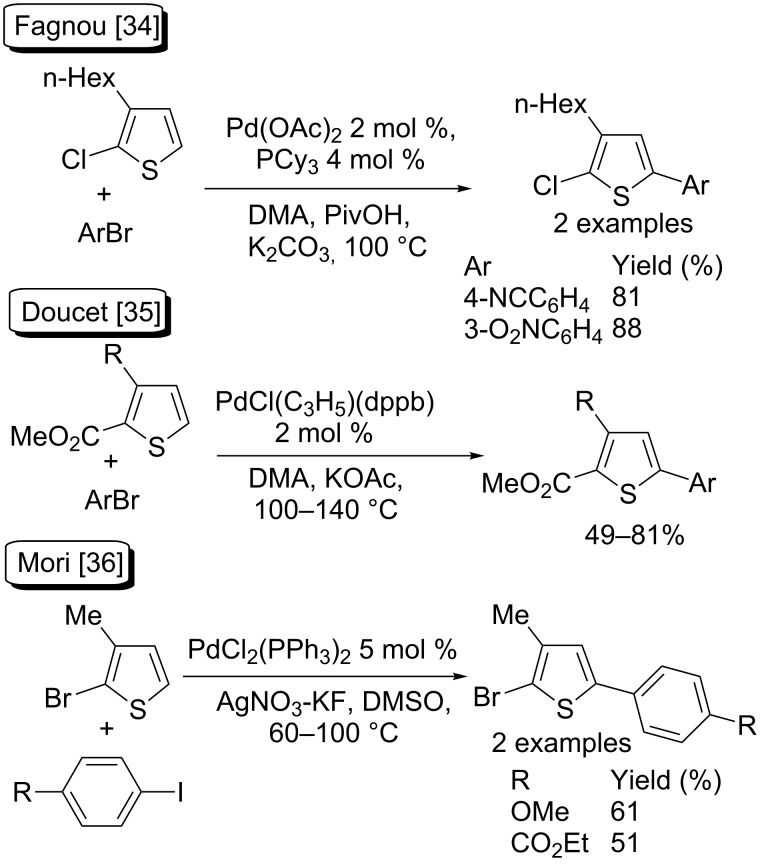
Blocking groups allowing regioselective C5-arylation of thiophenes.

In 2010, Fagnou et al*.* attached a 2-chloro-substituent to the thiophene ring to selectively perform a Pd-catalysed direct arylation of 3-hexylthiophene at the C5-position (Scheme 1, top) [34]. An ester moiety as blocking group at the C2-position of 3-substituted thiophene could also direct regioselectivity of Pd-catalysed direct arylation to the C5-position (Scheme 1, middle) [35]. Mori et al*.* also reported two examples of C5-arylation of 2-bromo-3-methylthiophene with aryl iodides as aryl sources with 5 mol % PdCl_2_(PPh_3_)_2_ catalyst and AgNO_3_–KF as the base in DMSO (Scheme 1, bottom) [36].

Herein, we wish to report on green conditions in terms of number of steps, base nature, use of a phosphine-free catalyst at low loading and a quite “atom economic” aryl source promoting such a C5-arylation using C3-substituted 2-bromothiophenes. We report i) that only 1 mol % of air-stable Pd(OAc)_2_ catalyst associated to KOAc promotes the regiospecific access to C5-arylated 2-bromothiophenes without cleavage of the thienyl C–Br bond, ii) on the reaction scope using a set of aryl bromides and 2-bromo-3-substituted thiophenes, iii) conditions allowing either the sequential C5-arylation followed by C2-arylation or C2-heteroarylation followed by C5-arylation of C3-substituted thiophenes.

## Results and Discussion

Based on some of our previous results on Pd-catalysed direct arylation, for this study, DMA and KOAc were selected as the solvent and base [35]. The reaction of 2 equiv of 2-bromothiophene with 1 equiv of 4-bromonitrobenzene using 1 mol % of phosphine-free Pd(OAc)_2_ catalyst performed at 110 °C, only afforded the desired product **1** in a trace amount, but a complete conversion of 2-bromothiophene was observed, revealing the high reactivity of the thienyl C–Br bond under these conditions (Table 1, entry 1). Using a lower reaction temperature of 80 °C, and a reaction time of 15 h, the desired C5-arylated product **1** was formed in only 8% yield due again to the formation of several degradation products (Table 1, entry 2). Then, we examined the influence of the reaction time. After 2 or 4 h, higher yields of **1** (55% and 48%) were obtained, respectively; whereas, a very short reaction time of 0.5 h led to a lower yield of 27% due to the poor conversion of 4-bromonitrobenzene (Table 1, entries 3–6). The use of 0.5 mol % Pd(OAc)_2_ catalyst at 80 °C during 2 h also afforded **1** in a lower yield of 35%. Again, a large amount of 4-bromonitrobenzene was recovered (Table 1, entry 7). When CsOAc, NaOAc or K_2_CO_3_ were employed as bases instead of KOAc, in the presence of 1 mol % Pd(OAc)_2_ catalyst during 2 h, a partial conversion of 4-bromonitrobenzene was observed and **1** was isolated in 32–40% yield (Table 1, entries 8–10). It should be noted that in the presence of cyclopentyl methyl ether or diethyl carbonate as solvents, no formation of **1** was observed, and 4-bromonitrobenzene was recovered unreacted (Table 1, entries 11 and 12).

**Table 1:** Influence of the reaction conditions for the palladium-catalysed direct C5-arylation of 2-bromothiophene with 4-bromonitrobenzene.^a^

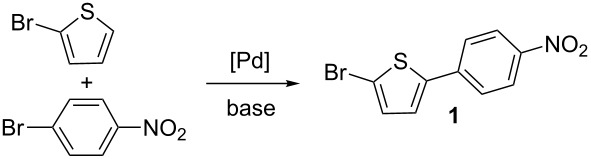					

Entry	Pd(OAc)_2_ (mol %)	Base	Temp (°C)	Time (h)	Yield in **1** (%)

1	1	KOAc	110	15	trace
2	1	KOAc	80	15	8
3	1	KOAc	80	4	48
4	1	KOAc	80	2	55
5	1	KOAc	80	1	42
6	1	KOAc	80	0.5	27
7	0.5	KOAc	80	2	35
8	1	CsOAc	80	2	35
9	1	NaOAc	80	2	32
10	1	K_2_CO_3_	80	2	40
11	1	KOAc	80	2	0^b^
12	1	KOAc	80	2	0^c^

^a^Conditions: Pd(OAc)_2_, 2-bromothiophene (2 equiv), 4-bromonitrobenzene (1 equiv), base (2 equiv), DMA, isolated yields. ^b^Cyclopentyl methyl ether as solvent. ^c^Diethyl carbonate as solvent.

**Scheme 2:**
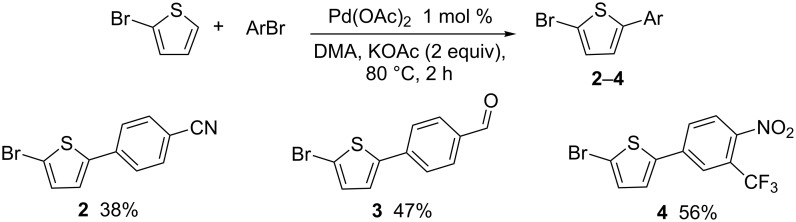
Reactivity of 2-bromothiophene with aryl bromides.

**Scheme 3:**
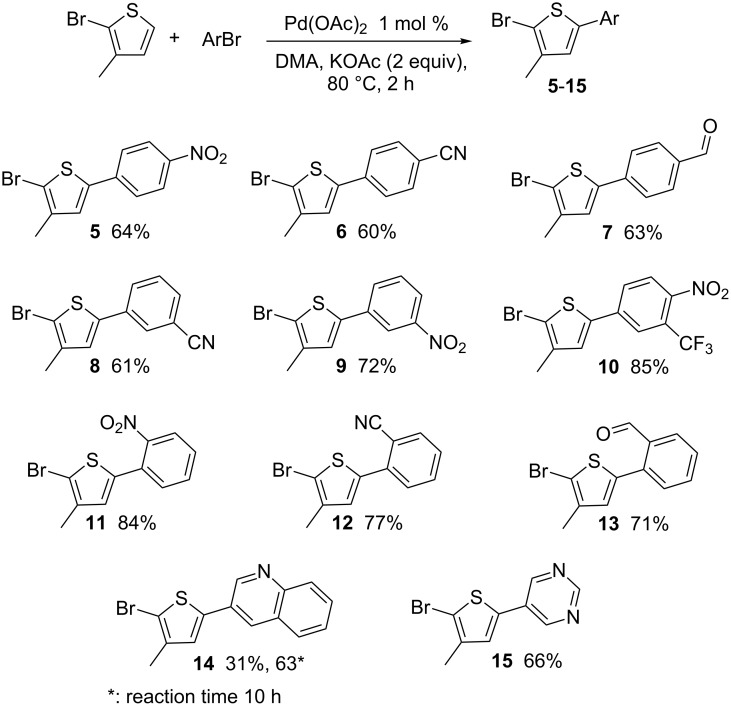
Reactivity of 2-bromo-3-methylthiophene with (hetero)aryl bromides.

**Scheme 4:**
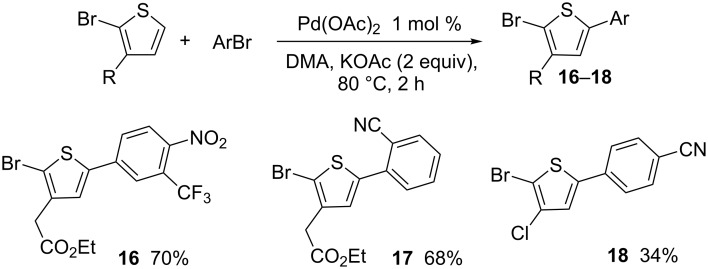
Reactivity of 3-substituted 2-bromothiophenes with aryl bromides.

Then, we studied the scope of this reaction using a set of aryl bromides and 2-bromothiophene derivatives, employing the most effective reaction conditions for C5-arylation of 2-bromothiophene (Table 1, entry 4: 1 mol % Pd(OAc)_2_, DMA, KOAc, 80 °C, 2 h) (Schemes 2–4). First, we investigated the reaction of 2-bromothiophene with 4-bromobenzonitrile, 4-bromobenzaldehyde and 4-bromo-2-(trifluoromethyl)nitrobenzene (Scheme 2). The expected coupling products **2–4** were obtained in moderate yields. On the other hand, with 4-bromoanisole as an electron-rich aryl bromide, the desired C5-arylated 2-bromothiophene could not be detected by GC–MS analysis of the crude mixture, and a large amount of unreacted 4-bromoanisole was recovered. Under these reaction conditions, the oxidative addition of 4-bromoanisole to palladium appears to be slower than the oxidative addition of 2-bromothiophene. Therefore, this procedure is limited to the use of electron-deficient aryl bromides. The reactivity of 2-bromofuran with 4-bromonitrobenzene was also investigated. Under the same reaction conditions, (1 mol % Pd(OAc)_2_, DMA, KOAc, 80 °C, 2 h) no formation of the desired 2-bromo-5-arylfuran derivative was observed.

The main interest to tolerate a C–Br bond at the C2-position on thiophene derivatives in the course of such couplings would be the regiospecific access to C5-arylated 3-substituted thiophenes, which cannot be obtained from 2-unsubstituted 3-substituted thiophenes such as 3-methylthiophene. Therefore, a set of aryl bromides was reacted with 2-bromo-3-methylthiophene, under these conditions (Scheme 3). Its reaction with aryl bromides *para*-substituted by nitro, cyano or formyl substituents gave the desired 5-arylated thiophenes **5**–**7** in 60–64% yields, without cleavage of the thienyl C–Br bond. Good yields of products **8** and **9** were also obtained from the *meta*-substituted aryl bromides, 3-bromobenzonitrile and 3-bromonitrobenzene. Again, a high yield of 85% of **10** was obtained with 4-bromo-2-(trifluoromethyl)nitrobenzene. Then, the reactivity of a set of *ortho*-substituted aryl bromides was examined. Bromobenzene containing nitro, nitrile or formyl *ortho*-substituents afforded the C5-arylated thiophenes **11**–**13** in 71–84% yields. Finally, 3-bromoquinoline and 3-bromopyrimidine were reacted with 2-bromo-3-methylthiophene affording **14** and **15** in 63% and 66% yields, respectively. The higher yields obtained for the arylation of 2-bromo-3-methylthiophene than with 2-bromothiophene are probably due to a slower oxidative addition of 2-bromo-3-methylthiophene to palladium which reduces the formation of side-products.

The reaction is not limited to the use of 2-bromo-3-methylthiophene. A 2-bromothiophene derivative bearing a CH_2_CO_2_Et substituent at C3 also provides regioselectively the desired C5-arylated thiophenes **16** and **17** in good yields; whereas, a lower yield of **18** was obtained for the coupling of 2-bromo-3-chlorothiophene with 4-bromobenzonitrile (Scheme 4).

**Scheme 5:**
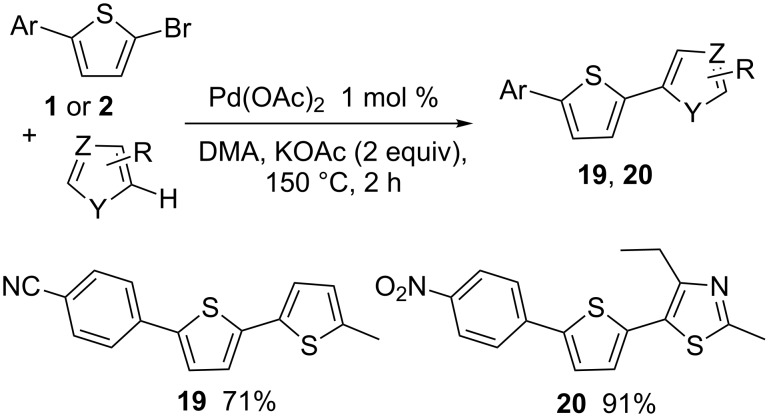
5-Heteroarylation of 2-aryl-5-bromothiophenes.

Then, to demonstrate the synthetic potential of the thienyl bromo-substituent, product **1** was coupled with 2-methylthiophene in the presence of 1 mol % Pd(OAc)_2_ catalyst and KOAc as base (Scheme 5). The desired product **19** was obtained in 71% yield. Under the same conditions, a high yield of 91% in **20** was obtained from **2** and 2-methyl-4-ethylthiazole. These two reactions demonstrate that the sequential Pd-catalysed direct di-(hetero)arylation, using 2-bromothiophene as central unit, provides a powerful and simple access to non-symmetrically 2,5-di(hetero)arylated thiophene derivatives.

**Scheme 6:**
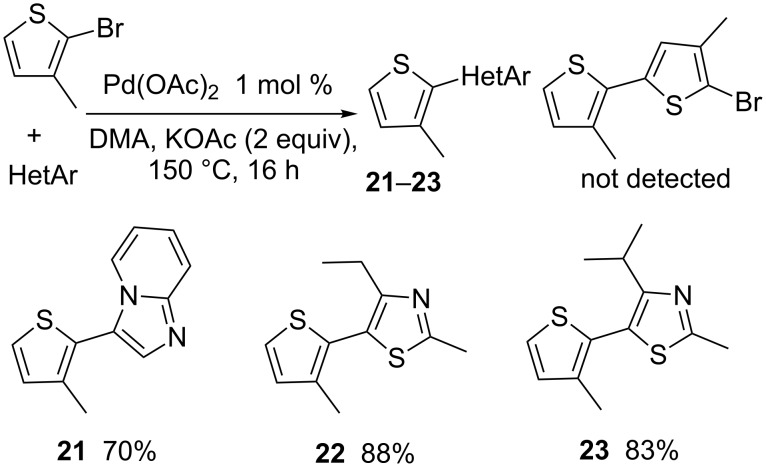
2-Heteroarylation of 2-bromo-3-methylthiophene.

**Scheme 7:**
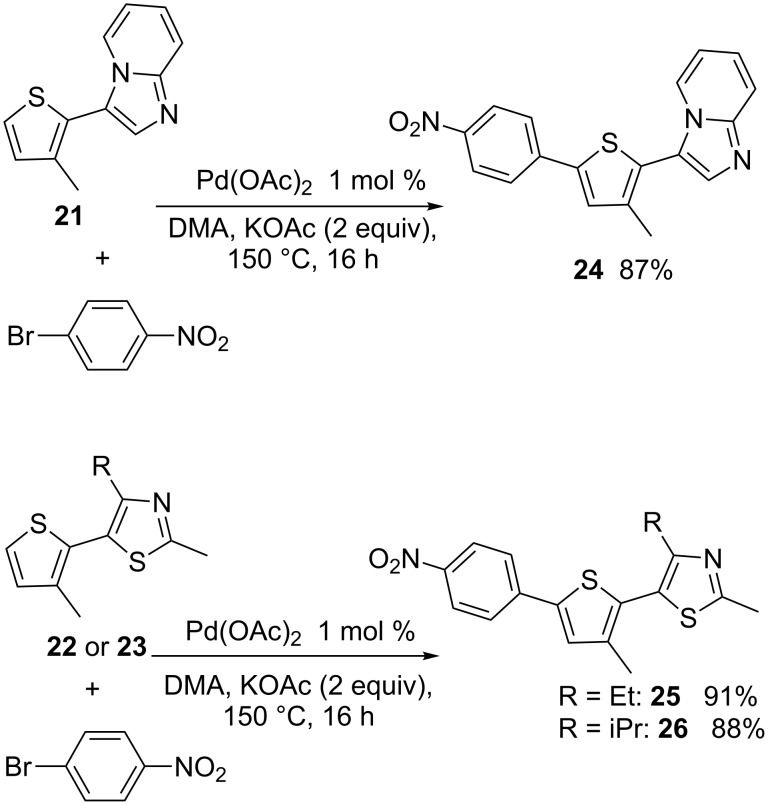
5-Arylation of 2,3-disubstituted thiophenes.

3-Substituted thiophene derivatives containing a heteroaryl unit at the C2-position and an aryl at C5 can also be obtained by direct heteroarylation at the C2-position of the C3-substituted 2-bromothiophene, followed by direct arylation at C5 (Scheme 6 and Scheme 7). First, we introduced imidazopyridinyl or thiazolyl groups at C2-position of 2-bromo-3-methylthiophene. In the presence of 1 mol % Pd(OAc)_2_ and KOAc as base at 150 °C the products **21**–**23** were obtained in 70–88% yields. In all cases, no C2-arylation of the 2-bromo-3-methylthiophene with itself to produce 5'-bromo-3,4'-dimethyl-2,2'-bithiophene was observed.

Then, from the C2-heteroarylated 3-methylthiophenes **21**–**23**, a second direct arylation at position C5 allows to prepare the products **24**–**26** in 87–91% yields (Scheme 7).

**Scheme 8:**
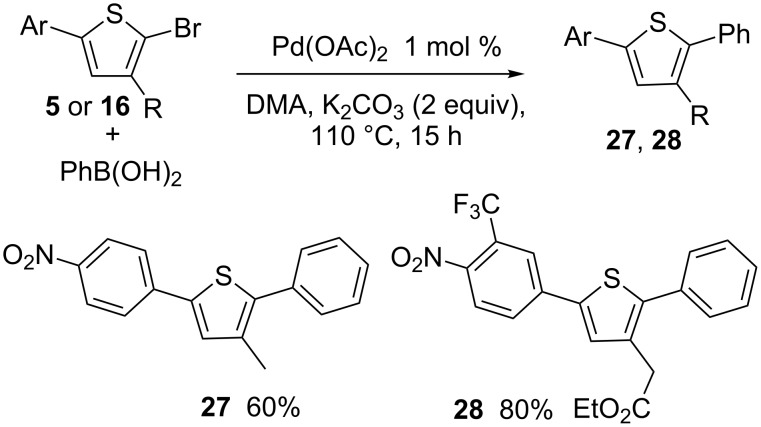
5-Arylation of 2-aryl-5-bromothiophenes.

The synthesis of 3-substituted thiophenes derivatives containing two different aryl groups at C2 and C5 positions via Suzuki coupling in the second step was also attempted (Scheme 8). The reaction of **5** with phenylboronic acid in the presence of only 1 mol % Pd(OAc)_2_ catalyst and K_2_CO_3_ as base gave 3-methyl-5-(4-nitrophenyl)-2-phenylthiophene (**27**) in 60% yield. A higher yield of 80% in **28** was obtained for the coupling of **16** with phenylboronic acid.

**Scheme 9:**
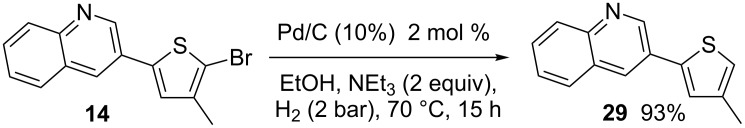
Deprotection of 2-aryl-5-bromothiophene **14**.

In order to further demonstrate that a bromo-substituent at C2-position of the thiophenes can be considered as a protecting group, we removed it via palladium-catalysed hydrogenolysis (Scheme 9). Treatment of **14** with 2 mol % Pd/C (10%) and trimethylamine in ethanol under hydrogen pressure, gave the desired debrominated product **29** in almost quantitative yield.

## Conclusion

In summary, we report here that the use of a 2-bromo-substituent on thiophenes acts as a blocking group, allowing their regioselective Pd-catalysed C5-arylation even in the presence of aryl bromides as aryl sources. Only 1 mol % of phosphine-free air stable Pd(OAc)_2_ catalyst in the presence of KOAc as base promotes the C5-arylation of 2-bromothiophenes containing various C3-substituents with electron-deficient (hetero)aryl bromides. The sequential direct C5-arylation of 2-bromothiophenes followed either by a Suzuki coupling or a second direct arylation was found to allow the preparation of 2,5-di(hetero)arylated thiophenes bearing two different (hetero)aryl units. This method provides a convenient “greener” access to arylated thiophene derivatives as 1) it reduces the number of steps to prepare these compounds, 2) it employs the easily available Pd(OAc)_2_ catalyst and aryl bromides as aryl sources, and the inexpensive base KOAc, 3) it reduces the formation of wastes.

## Supporting Information

File 1 Procedures, ^1^H and ^13^C NMR data of all compounds.
