# Supplementary material for: Regiocontroled Pd-catalysed C5-arylation of 3-substituted thiophene derivatives using a bromo-substituent as blocking group
Source: Beilstein J Org Chem. 2016 Oct 17;12:2197–203. doi: 10.3762/bjoc.12.210 (PMC5082451; doi:10.3762/bjoc.12.210)
Supplement: File 1 — Procedures, 1H and 13C NMR data of all compounds. [file Beilstein_J_Org_Chem-12-2197-s001.pdf]

Supporting Information

for

Regiocontroled Pd-catalysed C5-arylation of 3-substituted thiophene derivatives using a bromo-substituent as blocking group

Mariem Brahim[1,2], Hamed Ben Ammar[*2], Jean-François Soulé[*1] and Henri Doucet[*1]

Address: [1]Institut des Sciences Chimiques de Rennes, UMR 6226 CNRS-Université de Rennes "Organométalliques: Matériaux et Catalyse", Campus de Beaulieu, 35042 Rennes, France. Tel.: 00-33-2-23-23-63-84 and [2]Laboratoire de Synthèse Organique Asymétrique et Catalyse Homogène, (UR 11ES56) Université de Monastir, Faculté des Sciences de Monastir, avenue de l'environnement, Monastir 5000, Tunisie.

Email: Jean-François Soulé - jean-francois.soule@univ-rennes1.fr; Henri Doucet - henri.doucet@univ-rennes1.fr

*Corresponding author

Procedures, ^{1}H and ^{13}C NMR data of all compounds

All reactions were run under argon in Schlenk tubes using vacuum lines. DMA analytical grade was not distilled before use. KOAc (99%) and $Pd(OAc)_2$ (98%) were used. Commercial thiophene derivatives, aryl bromides and heteroarenes were used without purification. The reactions were followed by GC and NMR. ^{1}H and ^{13}C spectra were recorded with a Bruker 400 MHz spectrometer in $CDCl_3$ solutions. Chemical shifts are reported in ppm relative to $CDCl_3$ (7.25 for ^{1}H NMR and 77.0 for ^{13}C NMR). Flash chromatography was performed on silica gel (230–400 mesh).

General procedure for the synthesis of 1–18

In a similar manner as described in [1], as a typical experiment, the 2-bromothiophene derivative (2 mmol), aryl bromide derivative (1 mmol), KOAc (0.196 g, 2 mmol) and $Pd(OAc)_2$ (2.2 mg, 0.01 mmol) were dissolved in DMA (5 mL) under an argon atmosphere. The reaction mixture was stirred at 80 °C for 2 h. After evaporation of the solvent, the product was purified by silica gel column chromatography.

2-Bromo-5-(4-nitrophenyl)thiophene (1) [2]

From 2-bromothiophene (0.326 g, 2 mmol) and 4-bromonitrobenzene (0.202 g, 1 mmol) product **1** was obtained in 55% (0.156 g) yield as a yellow solid mp 130-132°C.

^{1}H NMR (400 MHz, $CDCl_3$) δ 8.23 (d, J = 8.9 Hz, 2H), 7.64 (d, J = 8.9 Hz, 2H), 7.22 (d, J = 4.0 Hz, 1H), 7.10 (d, J = 4.0 Hz, 1H).

^{13}C NMR (100 MHz, $CDCl_3$) δ 147.1, 143.2, 139.8, 131.8, 126.1, 126.0, 124.8, 115.1.

4-(5-Bromothiophen-2-yl)benzonitrile (2) [3]

From 2-bromothiophene (0.326 g, 2 mmol) and 4-bromobenzonitrile (0.182 g, 1 mmol) product **2** was obtained in 38% (0.100 g) yield as a yellow solid mp 102-104°C.

^{1}H NMR (400 MHz, $CDCl_3$) δ 7.65 (d, *J* = 8.5 Hz, 2H), 7.59 (d, *J* = 8.5 Hz, 2H), 7.16 (d, *J* = 4.0 Hz, 1H), 7.07 (d, *J* = 4.0 Hz, 1H).

^{13}C NMR (100 MHz, $CDCl_3$) δ 143.6, 138.0, 133.1, 131.6, 126.0, 125.6, 118.9, 114.4, 111.3.

4-(5-Bromothiophen-2-yl)benzaldehyde (3) [4]

From 2-bromothiophene (0.326 g, 2 mmol) and 4-bromobenzaldehyde (0.185 g, 1 mmol) product **3** was obtained in 47% (0.125 g) yield as a yellow solid mp 122-124°C.

^{1}H NMR (400 MHz, $CDCl_3$) δ 9.99 (s, 1H), 7.88 (d, *J* = 8.2 Hz, 2H), 7.65 (d, *J* = 8.2 Hz, 2H), 7.19 (d, *J* = 3.9 Hz, 1H), 7.07 (d, *J* = 3.9 Hz, 1H).

^{13}C NMR (100 MHz, $CDCl_3$) δ 191.6, 144.4, 139.4, 135.6, 131.6, 130.8, 126.0, 125.5, 114.2.

2-Bromo-5-(4-nitro-3-(trifluoromethyl)phenyl)thiophene (4)

From 2-bromothiophene (0.326 g, 2 mmol) and 4-bromo-1-nitro-2-(trifluoromethyl)benzene (0.270 g, 1 mmol) product **4** was obtained in 56% (0.197 g) yield as a yellow solid mp 94-96°C.

^{1}H NMR (400 MHz, $CDCl_3$) δ 7.95 (d, *J* = 8.5 Hz, 1H), 7.89 (s, 1H), 7.78 (dd, *J* = 8.5, 1.8 Hz, 1H), 7.24 (d, *J* = 3.9 Hz, 1H), 7.12 (d, *J* = 3.9 Hz, 1H).

^{13}C NMR (100 MHz, $CDCl_3$) δ 146.6, 141.6, 138.5, 132.0, 129.1, 126.8, 126.7, 125.9 (q, *J* = 34.0 Hz), 124.6 (q, *J* = 5.5 Hz), 122.0 (q, *J* = 273.8 Hz), 115.9.

$C_{11}H_5BrF_3NO_2S$ (352.13): Calcd C 37.52, H 1.43; Found C 37.34, H 1.37.

2-Bromo-3-methyl-5-(4-nitrophenyl)thiophene (5)

From 2-bromo-3-methylthiophene (0.354 g, 2 mmol) and 4-bromonitrobenzene (0.202 g, 1 mmol) product **5** was obtained in 64% (0.191 g) yield as a yellow solid mp 102-104°C.

^{1}H NMR (400 MHz, $CDCl_3$) δ 8.22 (d, *J* = 8.9 Hz, 2H), 7.62 (d, *J* = 8.9 Hz, 2H), 7.16 (s, 1H), 2.24 (s, 3H).

^{13}C NMR (100 MHz, $CDCl_3$) δ 146.5, 140.3, 139.6, 138.9, 127.3, 125.3, 124.3, 111.9, 15.2.

$C_{11}H_8BrNO_2S$ (298.15): Calcd C 44.31, H 2.70; Found C 44.50, H 2.99.

4-(5-Bromo-4-methylthiophen-2-yl)benzonitrile (6) [5]

From 2-bromo-3-methylthiophene (0.354 g, 2 mmol) and 4-bromobenzonitrile (0.182 g, 1 mmol) product **6** was obtained in 60% (0.167 g) yield as a yellow solid mp 82-84°C.

^{1}H NMR (400 MHz, $CDCl_3$) δ 7.61 (d, *J* = 8.6 Hz, 2H), 7.53 (d, *J* = 8.6 Hz, 2H), 7.07 (s, 1H), 2.20 (s, 3H).

^{13}C NMR (100 MHz, $CDCl_3$) δ 141.2, 139.1, 138.1, 133.0, 127.2, 125.7, 118.9, 111.7, 111.0, 15.6.

4-(5-Bromo-4-methylthiophen-2-yl)benzaldehyde (7)

From 2-bromo-3-methylthiophene (0.354 g, 2 mmol) and 4-bromobenzaldehyde (0.185 g, 1 mmol) product **7** was obtained in 63% (0.177 g) yield as a yellow solid mp 124-126°C.

^{1}H NMR (400 MHz, $CDCl_3$) δ 9.98 (s, 1H), 7.86 (d, J = 8.4 Hz, 2H), 7.63 (d, J = 8.4 Hz, 2H), 7.13 (s, 1H), 2.22 (s, 3H).

^{13}C NMR (100 MHz, $CDCl_3$) δ 191.9, 142.2, 139.9, 139.4, 135.8, 131.1, 127.4, 126.0, 111.8, 15.9.

$C_{12}H_9BrOS$ (281.17): Calcd C 51.26, H 3.23; Found C 51.40, H 3.17.

3-(5-Bromo-4-methylthiophen-2-yl)benzonitrile (8) [5]

From 2-bromo-3-methylthiophene (0.354 g, 2 mmol) and 3-bromobenzonitrile (0.182 g, 1 mmol) product **8** was obtained in 61% (0.169 g) yield as a yellow solid mp 128-130°C.

^{1}H NMR (400 MHz, $CDCl_3$) δ 7.75 (s, 1H), 7.68 (d, J = 7.9 Hz, 1H), 7.54 (d, J = 7.8 Hz, 1H), 7.46 (t, J = 7.8 Hz, 1H), 7.04 (s, 1H), 2.22 (s, 3H).

^{13}C NMR (100 MHz, $CDCl_3$) δ 140.7, 138.8, 135.1, 130.9, 130.0, 129.5, 128.7, 126.4, 118.5, 113.4, 110.6, 15.4.

2-Bromo-3-methyl-5-(3-nitrophenyl)thiophene (9)

From 2-bromo-3-methylthiophene (0.354 g, 2 mmol) and 3-bromonitrobenzene (0.202 g, 1 mmol) product **9** was obtained in 72% (0.214 g) yield as a yellow solid mp 138-140°C.

^{1}H NMR (400 MHz, $CDCl_3$) δ 8.34 (t, J = 2.0 Hz, 1H), 8.11 (ddd, J = 8.1, 2.2, 0.9 Hz, 1H), 7.78 (ddd, J = 7.8, 1.7, 0.9 Hz, 1H), 7.54 (t, J = 8.1 Hz, 1H), 7.13 (s, 1H), 2.24 (s, 3H).

^{13}C NMR (100 MHz, $CDCl_3$) δ 148.9, 140.6, 138.9, 135.5, 131.1, 130.1, 126.8, 122.2, 120.0, 110.8, 15.5.

$C_{11}H_8BrNO_2S$ (298.15): Calcd C 44.31, H 2.70; Found C 44.40, H 2.58.

2-Bromo-3-methyl-5-(4-nitro-3-(trifluoromethyl)phenyl)thiophene (10)

From 2-bromo-3-methylthiophene (0.354 g, 2 mmol) and 4-bromo-1-nitro-2-(trifluoromethyl)benzene (0.270 g, 1 mmol) product **10** was obtained in 85% (0.311 g) yield as a yellow solid mp 138-140°C.

^{1}H NMR (400 MHz, $CDCl_3$) δ 7.94 (d, J = 8.5 Hz, 1H), 7.88 (s, 1H), 7.75 (dd, J = 8.5, 1.8 Hz, 1H), 7.19 (s, 1H), 2.25 (s, 3H).

^{13}C NMR (100 MHz, $CDCl_3$) δ 146.8, 140.0, 139.5, 139.1, 129.1, 128.7, 127.0, 125.5 (q, J = 34.0 Hz), 124.7 (q, J = 5.5 Hz), 122.3 (q, J = 273.8 Hz), 113.5, 15.9.

$C_{12}H_7BrF_3NO_2S$ (366.15): Calcd C 39.36, H 1.93; Found C 39.21, H 2.01.

2-Bromo-3-methyl-5-(2-nitrophenyl)thiophene (11)

From 2-bromo-3-methylthiophene (0.354 g, 2 mmol) and 2-bromonitrobenzene (0.202 g, 1 mmol) product **11** was obtained in 84% (0.250 g) yield as a yellow solid mp 66-68°C.

^{1}H NMR (400 MHz, $CDCl_3$) δ 7.74 (d, J = 7.9 Hz, 1H), 7.56 (t, J = 7.8 Hz, 1H), 7.50-7.43 (m, 2H), 6.77 (s, 1H), 2.19 (s, 3H).

^{13}C NMR (100 MHz, $CDCl_3$) δ 149.8, 138.6, 137.0, 132.6, 132.5, 129.7, 129.5, 128.3, 124.6, 111.7, 15.8.

$C_{11}H_8BrNO_2S$ (298.15): Calcd C 44.31, H 2.70; Found C 44.21, H 2.94.

2-(5-Bromo-4-methylthiophen-2-yl)benzonitrile (12)

From 2-bromo-3-methylthiophene (0.354 g, 2 mmol) and 2-bromobenzonitrile (0.182 g, 1 mmol) product **12** was obtained in 77% (0.214 g) yield as a yellow solid mp 104-106°C.

^{1}H NMR (400 MHz, $CDCl_3$) δ 7.72 (d, *J* = 7.9 Hz, 1H), 7.58 (t, *J* = 7.8 Hz, 1H), 7.52 (d, *J* = 7.8 Hz, 1H), 7.39 (t, *J* = 7.8 Hz, 1H), 7.32 (s, 1H), 2.25 (s, 3H).

^{13}C NMR (100 MHz, $CDCl_3$) δ 138.9, 138.8, 137.1, 134.7, 133.3, 129.7, 129.5, 128.1, 118.9, 111.9, 109.9, 15.7.

$C_{12}H_8BrNS$ (278.17): Calcd C 51.81, H 2.90; Found C 51.78, H 2.78.

2-(5-Bromo-4-methylthiophen-2-yl)benzaldehyde (13)

From 2-bromo-3-methylthiophene (0.354 g, 2 mmol) and 2-bromobenzaldehyde (0.185 g, 1 mmol) product **13** was obtained in 71% (0.199 g) yield as a yellow solid mp 98-100°C.

^{1}H NMR (400 MHz, $CDCl_3$) δ 10.20 (s, 1H), 7.99 (d, *J* = 7.9 Hz, 1H), 7.61 (t, *J* = 7.8 Hz, 1H), 7.49 (d, *J* = 7.8 Hz, 1H), 7.48 (t, *J* = 7.8 Hz, 1H), 6.75 (s, 1H), 2.24 (s, 3H).

^{13}C NMR (100 MHz, $CDCl_3$) δ 191.5, 138.0, 137.9, 137.1, 133.9, 133.5, 131.3, 130.8, 128.3, 127.8, 111.0, 15.1.

$C_{12}H_9BrOS$ (281.17): Calcd C 51.26, H 3.23; Found C 51.17, H 3.30.

3-(5-Bromo-4-methylthiophen-2-yl)quinoline (14)

From 2-bromo-3-methylthiophene (0.354 g, 2 mmol) and 3-bromoquinoline (0.208 g, 1 mmol) product **14** was obtained in 63% (0.191 g) yield as a yellow solid mp 114-116°C.

^{1}H NMR (400 MHz, $CDCl_3$) δ 9.08 (s, 1H), 8.14 (s, 1H), 8.08 (d, *J* = 8.3 Hz, 1H), 7.80 (d, *J* = 8.0 Hz, 1H), 7.69 (t, *J* = 7.8 Hz, 1H), 7.55 (t, *J* = 7.8 Hz, 1H), 7.16 (s, 1H), 2.25 (s, 3H).

^{13}C NMR (100 MHz, $CDCl_3$) δ 148.4, 147.9, 140.4, 139.3, 131.5, 130.1, 129.9, 128.4, 127.9, 127.5, 126.8, 110.7, 15.9

$C_{14}H_{10}BrNS$ (304.20): Calcd C 55.28, H 3.31; Found C 55.07, H 3.17.

5-(5-Bromo-4-methylthiophen-2-yl)pyrimidine (15)

From 2-bromo-3-methylthiophene (0.354 g, 2 mmol) and 5-bromopyrimidine (0.159 g, 1 mmol) product **15** was obtained in 66% (0.168 g) yield as a yellow solid mp 92-94°C.

^{1}H NMR (400 MHz, $CDCl_3$) δ 9.11 (s, 1H), 8.84 (s, 2H), 7.24 (s, 1H), 2.24 (s, 3H).

^{13}C NMR (100 MHz, $CDCl_3$) δ 157.5, 153.1, 139.1, 135.5, 128.3, 127.3, 111.7, 15.4.

$C_9H_7BrN_2S$ (255.13): Calcd C 42.37, H 2.77; Found C 42.31, H2.67.

Ethyl 2-(2-bromo-5-(4-nitro-3-(trifluoromethyl)phenyl)thiophen-3-yl)acetate (16)

From ethyl 2-(2-bromothiophen-3-yl)acetate (0.498 g, 2 mmol) and 4-bromo-1-nitro-2-(trifluoromethyl)benzene (0.270 g, 1 mmol) product **16** was obtained in 70% (0.306 g) yield as a yellow oil.

^{1}H NMR (400 MHz, $CDCl_3$) δ 7.95 (d, *J* = 8.5 Hz, 1H), 7.90 (s, 1H), 7.78 (dd, *J* = 8.5, 1.8 Hz, 1H), 7.36 (s, 1H), 4.21 (q, *J* = 7.6 Hz, 2H), 3.66 (s, 2H), 1.30 (t, *J* = 7.6 Hz, 3H).

^{13}C NMR (100 MHz, $CDCl_3$) δ 169.2, 146.0, 139.2, 137.7, 135.3, 128.3, 127.2, 126.0, 125.5 (q, *J* = 34.0 Hz), 124.0 (q, *J* = 5.5 Hz), 121.3 (q, *J* = 273.8 Hz), 114.4, 60.9, 34.6, 13.7.

$C_{15}H_{11}BrF_3NO_4S$ (438.22): Calcd C 41.11, H 2.53; Found C 41.30, H 2.38.

Ethyl 2-(2-bromo-5-(2-cyanophenyl)thiophen-3-yl)acetate (17)

From ethyl 2-(2-bromothiophen-3-yl)acetate (0.498 g, 2 mmol) and 2-bromobenzonitrile (0.182 g, 1 mmol) product **17** was obtained in 68% (0.238 g) yield as a yellow oil.

^{1}H NMR (400 MHz, $CDCl_3$) δ 7.73 (d, *J* = 7.9 Hz, 1H), 7.60 (t, *J* = 7.8 Hz, 1H), 7.54 (d, *J* = 7.8 Hz, 1H), 7.46 (s, 1H), 7.39 (t, *J* = 7.8 Hz, 1H), 4.21 (q, *J* = 7.6 Hz, 2H), 3.67 (s, 2H), 1.29 (t, *J* = 7.6 Hz, 3H).

^{13}C NMR (100 MHz, $CDCl_3$) δ 170.2, 139.7, 137.0, 135.4, 134.9, 133.5, 129.8, 129.4, 128.5, 118.9, 114.2, 110.4, 61.8, 35.7, 14.7.

$C_{15}H_{12}BrNO_2S$ (350.23): Calcd C 51.44, H 3.45; Found C 51.38, H 3.37.

4-(5-Bromo-4-chlorothiophen-2-yl)benzonitrile (18)

From 2-bromo-3-chlorothiophene (0.394 g, 2 mmol) and 4-bromobenzonitrile (0.182 g, 1 mmol) product **18** was obtained in 34% (0.101 g) yield as a yellow solid mp 158-160°C.

^{1}H NMR (400 MHz, $CDCl_3$) δ 7.68 (d, *J* = 8.0 Hz, 2H), 7.58 (d, *J* = 8.0 Hz, 2H), 7.19 (s, 1H).

^{13}C NMR (100 MHz, $CDCl_3$) δ 141.8, 137.0, 133.1, 128.5, 125.8, 125.2, 118.5, 112.0, 110.3.

$C_{11}H_5BrClNS$ (298.59): Calcd C 44.25, H 1.69; Found C 44.36, H 1.78.

4-(5'-Methyl-2,2'-bithiophen-5-yl)benzonitrile (19)

In a similar manner as described in [1], 4-(5-bromothiophen-2-yl)benzonitrile (**2**, 0.264 g, 1 mmol), 2-methylthiophene (0.049 g, 0.5 mmol), KOAc (0.098 g, 1 mmol) and $Pd(OAc)_2$ (1.1 mg, 0.005 mmol), were dissolved in DMA (3 mL) under an argon atmosphere. The reaction mixture was stirred at 150 °C for 2 h. After evaporation of the solvent, the product was purified by silica gel column chromatography affording **19** in 71% (0.100 g) yield as a yellow solid mp 178-180°C.

^{1}H NMR (400 MHz, $CDCl_3$) δ 7.68-7.60 (m, 4H), 7.31 (d, *J* = 3.5 Hz, 1H), 7.08 (d, *J* = 3.5 Hz, 1H), 7.02 (d, *J* = 3.5 Hz, 1H), 6.69 (d, *J* = 3.5 Hz, 1H), 2.50 (s, 3H).

^{13}C NMR (100 MHz, $CDCl_3$) δ 140.8, 140.3, 140.2, 139.0, 134.9, 133.3, 126.7, 126.4, 126.3, 124.8, 124.7, 119.4, 110.8, 16.0.

$C_{16}H_{11}NS_2$ (281.39): Calcd C 68.29, H 3.94; Found C 68.21, H 3.80.

4-Ethyl-2-methyl-5-(5-(4-nitrophenyl)thiophen-2-yl)thiazole (20)

In a similar manner as described in [1], 2-bromo-5-(4-nitrophenyl)thiophene (**1,** 0.284 g, 1 mmol), 2-ethyl-4-methylthiazole (0.064 g, 0.5 mmol), KOAc (0.098 g, 1 mmol) and $Pd(OAc)_2$ (1.1 mg, 0.005 mmol), were dissolved in DMA (3 mL) under an argon atmosphere. The reaction mixture was stirred at 150 °C for 2 h. After evaporation of the solvent, the product was purified by silica gel column chromatography affording **20** in 91% (0.150 g) yield as a yellow solid mp 118-120°C.

^{1}H NMR (400 MHz, $CDCl_3$) δ 8.25 (d, *J* = 8.4 Hz, 2H), 7.73 (d, *J* = 8.4 Hz, 2H), 7.43 (d, *J* = 3.7 Hz, 1H), 7.11 (d, *J* = 3.7 Hz, 1H), 3.00 (q, *J* = 7.6 Hz, 2H), 2.59 (s, 3H), 1.40 (t, *J* = 7.6 Hz, 3H).

^{13}C NMR (100 MHz, $CDCl_3$) δ 171.6, 149.5, 147.6, 142.1, 141.0, 137.4, 128.7, 127.1, 126.7, 125.5, 124.9, 27.9, 17.8, 15.1.

$C_{16}H_{14}N_2O_2S_2$ (330.42): Calcd C 58.16, H 4.27; Found C 58.24, H 4.19.

General procedure for the synthesis of 21–23

In a similar manner as described in [1], as a typical experiment, the 2-bromothiophene derivative (1 mmol), heteroarene derivative (2 mmol), KOAc (0.196 g, 2 mmol) and $Pd(OAc)_2$ (2.2 mg, 0.01 mmol), were dissolved in DMA (5 mL) under an argon atmosphere. The reaction mixture was stirred at 150 °C for 16 h. After evaporation of the solvent, the product was purified by silica gel column chromatography.

3-(3-Methylthiophen-2-yl)imidazo[1,2-a]pyridine (21)

From 2-bromo-3-methylthiophene (0.177 g, 1 mmol) and imidazo[1,2-a]pyridine (0.236 g, 2 mmol) product **21** was obtained in 70% (0.150 g) yield as a yellow oil.

^{1}H NMR (400 MHz, $CDCl_3$) δ 7.97 (d, J = 6.9 Hz, 1H), 7.65 (s, 1H), 7.64 (d, J = 8.7 Hz, 1H), 7.37 (d, J = 5.2 Hz, 1H), 7.18 (dd, J = 8.7, 6.9 Hz, 1H), 7.00 (d, J = 5.2 Hz, 1H), 6.78 (t, J = 6.9 Hz, 1H), 2.11 (s, 3H).

^{13}C NMR (100 MHz, $CDCl_3$) δ 146.3, 138.6, 134.8, 130.6, 126.7, 124.9, 124.3, 123.4, 118.2, 117.8, 112.8, 14.9.

$C_{12}H_{10}N_2S$ (214.29): Calcd C 67.26, H 4.70; Found C 67.04, H 4.61.

4-Ethyl-2-methyl-5-(3-methylthiophen-2-yl)thiazole (22)

From 2-bromo-3-methylthiophene (0.177 g, 1 mmol) and 2-ethyl-4-methylthiazole (0.256 g, 2 mmol) product **22** was obtained in 88% (0.196 g) yield as a yellow oil.

^{1}H NMR (400 MHz, $CDCl_3$) δ 7.27 (d, J = 5.2 Hz, 1H), 6.91 (d, J = 5.2 Hz, 1H), 2.99 (q, J = 7.6 Hz, 2H), 2.32 (s, 3H), 2.16 (s, 3H), 1.39 (t, J = 7.6 Hz, 3H).

^{13}C NMR (100 MHz, $CDCl_3$) δ 172.1, 150.4, 137.4, 130.3, 127.1, 125.7, 122.6, 27.3, 16.1, 14.9, 14.4.

$C_{11}H_{13}NS_2$ (223.36): Calcd C 59.15, H 5.87; Found C 59.07, H 5.88.

4-Isopropyl-2-methyl-5-(3-methylthiophen-2-yl)thiazole (23)

From 2-bromo-3-methylthiophene (0.177 g, 1 mmol) and 4-isopropyl-2-methylthiazole (0.282 g, 2 mmol) product **21** was obtained in 83% (0.197 g) yield as a yellow oil.

^{1}H NMR (400 MHz, $CDCl_3$) δ 7.27 (d, J = 5.2 Hz, 1H), 6.91 (d, J = 5.2 Hz, 1H), 3.26 (sept., J = 7.6 Hz, 1H), 2.32 (s, 3H), 2.17 (s, 3H), 1.41 (d, J = 7.6 Hz, 6H).

^{13}C NMR (100 MHz, $CDCl_3$) δ 177.2, 150.3, 137.3, 130.3, 127.2, 125.7, 122.2, 33.7, 23.4, 16.2, 14.9.

$C_{12}H_{15}NS_2$ (237.38): Calcd C 60.72, H 6.37; Found C 60.77, H 6.20.

General procedure for the synthesis of 24–26

In a similar manner as described in [1], as a typical experiment, the thiophene derivative (1 mmol), aryl bromide derivative (1.5 mmol), KOAc (0.196 g, 2 mmol) and $Pd(OAc)_2$ (2.2 mg, 0.01 mmol), were dissolved in DMA (5 mL) under an argon atmosphere. The reaction mixture was stirred at 150 °C for 16 h. After evaporation of the solvent, the product was purified by silica gel column chromatography.

3-(3-Methyl-5-(4-nitrophenyl)thiophen-2-yl)imidazo[1,2-a]pyridine (24)

From 3-(3-methylthiophen-2-yl)imidazo[1,2-a]pyridine (**21**, 0.214 g, 1 mmol) and 4-bromonitrobenzene (0.303 g, 1.5 mmol) product **24** obtained in 87% (0.291 g) yield as a yellow solid mp 204-206°C.

^{1}H NMR (400 MHz, $CDCl_3$) δ 8.24 (d, J = 8.7 Hz, 2H), 8.13 (d, J = 6.9 Hz, 1H), 7.78-7.70 (m, 4H), 7.43 (s, 1H), 7.31-7.27 (m, 1H), 6.90 (t, J = 6.9 Hz, 1H), 2.22 (s, 3H).

^{13}C NMR (100 MHz, $CDCl_3$) δ 147.2, 146.7, 141.8, 140.3, 140.0, 135.4, 129.1, 126.1, 125.3, 124.8, 124.3, 118.5, 117.3, 113.3, 15.3.

$C_{18}H_{13}N_3O_2S$ (335.38): Calcd C 64.46, H 3.91; Found C 64.37, H 3.81.

4-Ethyl-2-methyl-5-(3-methyl-5-(4-nitrophenyl)thiophen-2-yl)thiazole (25)

From 4-ethyl-2-methyl-5-(3-methylthiophen-2-yl)thiazole (**22**, 0.223 g, 1 mmol) and 4-bromonitrobenzene (0.303 g, 1.5 mmol) product **25** was obtained in 91% (0.313 g) yield as a yellow oil.

^{1}H NMR (400 MHz, $CDCl_3$) δ 8.22 (d, J = 8.4 Hz, 2H), 7.69 (d, J = 8.4 Hz, 2H), 7.30 (s, 1H), 3.02 (q, J = 7.6 Hz, 2H), 2.38 (s, 3H), 2.21 (s, 3H), 1.41 (t, J = 7.6 Hz, 3H).

^{13}C NMR (100 MHz, $CDCl_3$) δ 173.0, 151.2, 147.2, 141.3, 140.7, 139.3, 130.2, 129.2, 126.2, 125.0, 122.2, 27.6, 16.7, 15.5, 14.7.

$C_{17}H_{16}N_2O_2S_2$ (344.45): Calcd C 59.28, H 4.68; Found C 59.20, H 4.40.

4-Isopropyl-2-methyl-5-(3-methyl-5-(4-nitrophenyl)thiophen-2-yl)thiazole (26)

From 4-isopropyl-2-methyl-5-(3-methylthiophen-2-yl)thiazole (**23**, 0.237 g, 1 mmol) and 4-bromonitrobenzene (0.303 g, 1.5 mmol) product **26** was obtained in 88% (0.315 g) yield as a yellow solid mp 100-102°C.

^{1}H NMR (400 MHz, $CDCl_3$) δ 8.23 (d, J = 8.9 Hz, 2H), 7.70 (d, J = 8.9 Hz, 2H), 7.31 (s, 1H), 3.29 (sept., J = 7.6 Hz, 1H), 2.39 (s, 3H), 2.22 (s, 3H), 1.42 (d, J = 7.6 Hz, 6H).

^{13}C NMR (100 MHz, $CDCl_3$) δ 177.8, 150.8, 146.9, 141.0, 140.4, 139.0, 130.1, 128.9, 125.9, 124.7, 121.5, 33.7, 23.4, 16.5, 15.2.

$C_{18}H_{18}N_2O_2S_2$ (358.48): Calcd C 60.31, H 5.06; Found C 60.24, H 5.02.

3-Methyl-5-(4-nitrophenyl)-2-phenylthiophene (27)

2-Bromo-3-methyl-5-(4-nitrophenyl)thiophene (**5**, 0.149 g, 0.5 mmol), phenylboronic acid (0.092 g, 0.75 mmol), K_2CO_3 (0.138 g, 1 mmol) and $Pd(OAc)_2$ (1.1 mg, 0.005 mmol), were dissolved in DMA (3 mL) under an argon atmosphere. The reaction mixture was stirred at 110 °C for 15h. After evaporation of the solvent, the product was purified by silica gel column chromatography affording **27** in 60% (0.088 g) yield as a yellow solid mp 100-102°C.

^{1}H NMR (400 MHz, $CDCl_3$) δ 8.23 (d, J = 8.9 Hz, 2H), 7.72 (d, J = 8.9 Hz, 2H), 7.51 (d, J = 8.0 Hz, 2H), 7.45 (t, J = 8.0 Hz, 2H), 7.35 (t, J = 8.0 Hz, 1H), 7.33 (s, 1H), 2.37 (s, 3H).

^{13}C NMR (100 MHz, $CDCl_3$) δ 146.6, 140.9, 140.7, 138.9, 135.1, 134.1, 129.9, 129.0, 128.9, 128.0, 125.6, 124.6, 15.3.

$C_{17}H_{13}NO_2S$ (295.35): Calcd C 69.13, H 4.44; Found C 69.00, H 4.51.

Ethyl 2-(5-(4-nitro-3-(trifluoromethyl)phenyl)-2-phenylthiophen-3-yl)acetate (28)

Ethyl 2-(2-bromo-5-(4-nitro-3-(trifluoromethyl)phenyl)thiophen-3-yl)acetate (**16**, 0.219 g, 0.5 mmol), phenylboronic acid (0.092 g, 0.75 mmol), K_2CO_3 (0.138 g, 1 mmol) and $Pd(OAc)_2$ (1.1 mg, 0.005 mmol), were dissolved in DMA (3 mL) under an argon atmosphere. The reaction mixture was stirred at 110 °C for 15h. After evaporation of the solvent, the product was purified by silica gel column chromatography affording **28** in 80% (0.174 g) yield as a yellow solid mp 98-100°C.

^{1}H NMR (400 MHz, $CDCl_3$) δ 8.00 (s, 1H), 7.97 (d, *J* = 8.5 Hz, 1H), 7.86 (dd, *J* = 8.5, 1.8 Hz, 1H), 7.54-7.40 (m, 6H), 4.20 (q, *J* = 7.6 Hz, 2H), 3.68 (s, 2H), 1.28 (t, *J* = 7.6 Hz, 3H).

^{13}C NMR (100 MHz, $CDCl_3$) δ 171.1, 146.2, 144.1, 139.2, 138.2, 132.9, 131.5, 129.4, 129.2, 129.1, 128.9, 128.8, 126.5, 125.0 (q, *J* = 34.0 Hz), 124.6 (q, *J* = 5.5 Hz), 122.0 (q, *J* = 273.6 Hz), 61.4, 34.7, 14.3.

$C_{21}H_{16}F_3NO_4S$ (435.42): Calcd C 57.93, H 3.70; Found C 57.79, H 3.48.

3-(4-Methylthiophen-2-yl)quinoline (29)

3-(5-Bromo-4-methylthiophen-2-yl)quinoline (**14**, 0.152 g, 0.5 mmol), NEt_3 (0.101 g, 1 mmol) and Pd/C (10%) (10.6 mg, 0.01 mmol), were dissolved in EtOH (3 mL) under hydrogen atmosphere (2 bars). The reaction mixture was stirred at 70 °C for 15h. After evaporation of the solvent, the product was purified by silica gel column chromatography affording **29** in 93% (0.105 g) yield as a yellow solid mp 76-79°C.

^{1}H NMR (400 MHz, $CDCl_3$) δ 9.17 (d, *J* = 1.8 Hz, 1H), 8.22 (d, *J* = 1.8 Hz, 1H), 8.09 (d, *J* = 8.3 Hz, 1H), 7.81 (d, *J* = 8.0 Hz, 1H), 7.68 (t, *J* = 7.8 Hz, 1H), 7.54 (t, *J* = 7.8 Hz, 1H), 7.30 (s, 1H), 6.96 (s, 1H), 2.25 (s, 3H).

^{13}C NMR (100 MHz, $CDCl_3$) δ 148.7, 147.5, 140.6, 139.4, 131.3, 129.6, 129.5, 128.2, 128.1, 128.0, 127.5, 127.0, 121.7, 16.1.

$C_{14}H_{11}NS$ (225.31): Calcd C 74.63, H 4.92; Found C 74.75, H 4.98.

References

[1] Belkessam, F.; Mohand, A.; Soulé, J.-F.; Elias, A.; Doucet, H. *Beilstein J. Org. Chem.* **2014**, *10*, 2912-2919.

[2] El Bakouri, O.; Fernandez, M.; Brun, S.; Pla-Quintana, A.; Roglans, A. *Tetrahedron* **2013**, *69*, 9761-9765.

[3] Chaires, J. B.; Ren, J.; Hamelberg, D.; Kumar, A.; Pandya, V.; Boykin, D. W.; Wilson, W. D. *J. Med. Chem.* **2004**, *47*, 5729-5742.

[4] Scrascia, A.; De Marco, L.; Laricchia, S.; Picca, R. A.; Carlucci, C.; Fabiano, E.; Capodilupo, A. L.; Della Sala, F.; Gigli, G.; Ciccarella, G. *J. Mater. Chem. A* **2013**, *1*, 11909-11921.

[5] Kobayashi, K.; Sugie, A.; Takahashi, M.; Masui, K.; Mori, A. *Org. Lett.* **2005**, *7*, 5083-5085.
